# Automated Food Weight and Content Estimation Using Computer Vision and AI Algorithms: Phase 2

**DOI:** 10.3390/s26010076

**Published:** 2025-12-22

**Authors:** Bryan Gonzalez, Gonzalo Garcia, Sergio A. Velastin, Hamid GholamHosseini, Lino Tejeda, Heilym Ramirez, Gonzalo Farias

**Affiliations:** 1 Escuela de Ingeniería Eléctrica, Pontificia Universidad Católica de Valparaíso, Valparaíso 2362804, Chile; bryan.gonzalez.l@mail.pucv.cl (B.G.); gonzalo.farias@pucv.cl (G.F.); 2 College of Engineering, Virginia Commonwealth University, Richmond, VA 23284, USA; garciaga3@vcu.edu; 3 School of Electronic Engineering and Computer Science, Queen Mary University of London, London E1 4NS, UK; 4 Department of Computer Engineering, Universidad Carlos III de Madrid, 28903 Getafe, Spain; 5 School of Engineering, Computer and Mathematical Sciences, Auckland University of Technology, Auckland 1010, New Zealand; hamid.gholamhosseini@aut.ac.nz; 6 Sourcing, Santiago 7550000, Chile; ltejeda@sourcing.cl; 7 Departamento de Electrotecnia e Informática, Universidad Técnica Federico Santa María, Viña del Mar 2520000, Chile; heilym.ramirez@usm.cl

**Keywords:** food weight estimation, deep learning, computer vision, artificial intelligence

## Abstract

The work aims to leverage computer vision and artificial intelligence technologies to quantify key components in food catering services. Specifically, it focuses on content identification and portion size estimation in a dining hall setting, typical of corporate and educational settings. An RGB camera is employed to capture the tray delivery process in a self-service restaurant, providing test images for content identification algorithm comparison, using standard evaluation metrics. The approach utilizes the YOLO architecture, a widely recognized deep learning model for object detection and computer vision. The model is trained on labeled image data, and its performance is assessed using a precision–recall curve at a confidence threshold of 0.5, achieving a mean Average Precision (mAP) of 0.873, indicating robust overall performance. The weight estimation procedure combines computer vision techniques to measure food volume using both RGB and depth cameras. Subsequently, density models specific to each food type are applied to estimate the detected food weight. The estimation model’s parameters are calibrated through experiments that generate volume-to-weight conversion tables for different food items. Validation of the system was conducted using rice and chicken, yielding error margins of 5.07% and 3.75%, respectively, demonstrating the feasibility and accuracy of the proposed method.

## 1. Introduction

Accurate food quantification in institutional dining environments plays a critical role in improving food safety, minimizing waste, and ensuring compliance with dietary requirements. Traditional manual approaches to estimating food intake or serving portions are often error prone and labor intensive, especially in high-throughput settings such as hospitals, schools, or corporate cafeterias. These limitations motivate the development of automated, scalable solutions capable of detecting, identifying, and quantifying food items in real time [1,2,3,4,5].

A significant challenge lies in the lack of affordable and accurate depth-sensing solutions from practical overhead sensor installations [6]. Furthermore, there is a clear demand for improved generalization and precision in segmenting multi-ingredient meals, as prior models have struggled with complex clutter [7,8]. Ensuring proportionality between estimated volume and actual food mass is also crucial for accurate nutritional tracking [1,2]. Ultimately, the goal is to design a scalable system that performs robustly under diverse presentation and lighting conditions [9].

In this paper, we build upon the core principles of image-based analysis and AI-driven interpretation and apply them to the domain of food tray monitoring, where similar challenges arise, such as occlusion, varying presentation, and cluttered scenes.

This work presents a robust pipeline for ingredient-level food quantification using monocular RGB images. It combines state-of-the-art object detection and segmentation models with monocular depth estimation to infer the volume of food items and subsequently estimate their weight.

The key contributions of this paper are as follows:

- A full pipeline that combines YOLO-based ingredient detection [10] with SAM-based segmentation [11] for per-ingredient isolation.
- The use of monocular depth estimation via Depth Anything v2 [6] with ground-plane correction for accurate 3D volume measurement.
- An automated weight estimation method demonstrating errors <5.1% tested on real cafeteria items [9].
- A methodology designed for robustness under variable conditions, without the need for camera calibration [6,12].
- Integration of scalable, open-source tools suited for real-world deployment in institutions [10,11].

Unlike traditional inverse calibration techniques or stereo setups [13], our approach leverages recent advances in AI vision models to enhance accuracy, adaptability, and generalization in real-world conditions. The system is validated in controlled experiments using common cafeteria items (e.g., rice and chicken), with trials evaluating rotational invariance and consistency across repetitions. Results show strong agreement between estimated and actual weights, confirming the system’s feasibility as a practical and low-cost solution for automated food monitoring.

There are algorithms for object detection in images using both supervised and unsupervised approaches, including methods that focus on localizing object parts [4,5]. For example, PEEKABOO introduces an unsupervised strategy for object localization by selectively hiding image regions to improve part-based understanding. In contrast, our work adopts a supervised approach using YOLO due to its robustness and proven performance in prior studies for detecting trays, plates, and ingredients in institutional dining settings. This choice builds on our previous contributions and ensures reliable detection under real-world conditions.

The paper is structured as follows.

Section 2 describes the materials, hardware setup, and methodological framework used in this study, detailing the RGB acquisition system, monocular depth estimation model, and the proposed processing pipeline for segmentation and volume estimation. Section 3 presents the experimental results obtained from controlled cafeteria environments, including quantitative and qualitative evaluations of detection accuracy, depth consistency, and weight estimation performance. Finally, Section 4 concludes the paper, summarizing key findings and outlining directions for future work, such as real-time deployment and large-scale validation in institutional settings.

## 2. Materials and Methods

### 2.1. RGB Setup

The primary RGB acquisition in this study was performed using a Hikvision DS-2CD2786G2-IZS camera [14], mounted in a fixed overhead position approximately 2 m above the food tray. This setup aimed to simulate real-world deployment scenarios in institutional and corporate dining halls, where cameras cannot be placed at close distances due to safety or infrastructure constraints. The high-resolution and adjustable field of view of this camera make it a suitable candidate for detecting and segmenting food components over a large working area.

Table 1 summarizes the main technical specifications of the camera, which include an 8 MP sensor with a wide dynamic range (WDR), infrared capability for low-light conditions, and IP66/IK10 protection ratings for environmental robustness.

### 2.2. Monocular Depth Estimation Setup

A major methodological shift from previous stereo-based approaches was the adoption of monocular depth estimation using the Depth Anything V2 model [6]. In earlier phases of this research, depth estimation was carried out using the Luxonis OAK-D Pro, a stereo-based RGB-D camera positioned 40 cm above the tray. This setup provided accurate results for close-range captures.

**Figure 1:**
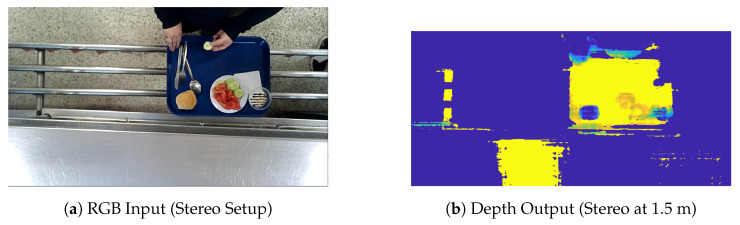
Example of stereo-based depth estimation failure using the OAK-D Lite camera at 1.5 m. The depth map (on the right) lacks structural fidelity, with severe flattening over the food area (the pseudo colors on the depth map represent depth and illustrate that it is difficult to separate the food items from the tray).

However, when the camera was repositioned to a more practical height of 2.0 m—due to safety, infrastructure, and deployment requirements, the quality of the stereo depth maps deteriorated significantly. Specifically, the upper surfaces of food items appeared flattened or poorly defined, with a noticeable loss of structure and depth continuity. This is illustrated in Figure 1.

Furthermore, replacing the OAK-D Lite with an industrial-grade stereo or LiDAR-based system that could maintain high accuracy at this distance would have resulted in hardware costs exceeding USD 20,000, making it economically unfeasible for scalable deployment in institutional or corporate food environments.

To overcome these limitations, we transitioned to a monocular depth estimation strategy using Depth Anything V2, a vision transformer trained on large-scale synthetic and real-world datasets. This approach enables the generation of dense depth maps from single RGB inputs, offering a cost-effective alternative with the flexibility to adapt to varying tray configurations and lighting conditions typical in food service environments.

The model introduces three key innovations that enhance depth estimation accuracy and generalization:

1. Training with highly accurate synthetic datasets instead of noisy real-world annotations.
2. Scaling up the teacher network using a DINOv2-G backbone for better pseudo-label generation.
3. Refining student networks with massive collections of real-world unlabeled images via distillation.

It is particularly robust in preserving fine object details (e.g., edges of thin or semi-transparent foods), which is essential for precise segmentation and downstream volume estimation. Its public availability via HuggingFace ensures reproducibility and eases integration into real-time systems.

For this study, the ViT-L (Large) variant was selected due to its balance between computational efficiency and depth quality. According to its authors, the model achieves 97.1% accuracy in relative depth ordering on the DA-2K benchmark, outperforming prior monocular methods across datasets such as NYU-D and KITTI.

Each RGB frame is preprocessed prior to inference. First, the tray bounding box is detected and used to crop the region of interest (ROI) from the full image. The cropped image is resized to 512 × 512 pixels, as required by the model. The output is a float32-format depth map with normalized relative values between 0 and 1, capturing spatial variation in elevation without metric calibration.

**Figure 2:**
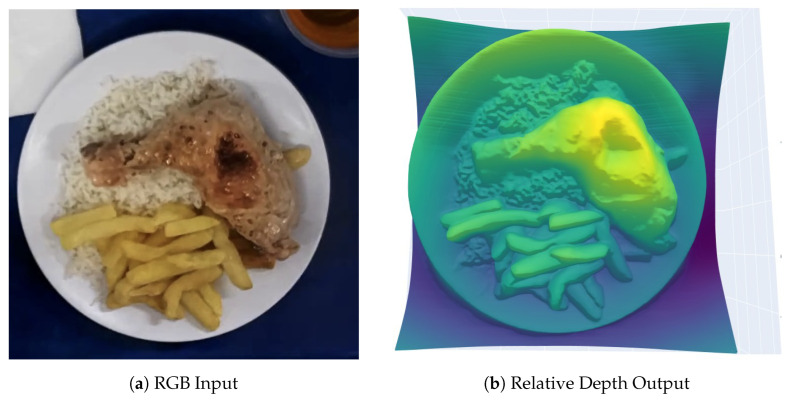
RGB and estimated depth map produced by Depth Anything V2. Depth is encoded as relative values between 0 and 1.

An example of an RGB input and the corresponding depth map generated by Depth Anything V2 is shown in Figure 2.

Although initially chosen for its implementation simplicity, Depth Anything V2 demonstrates performance that justifies its use beyond convenience alone. Table 2 compares its Absolute Relative Error (AbsRel) on two standard benchmarks—NYU Depth v2 and KITTI—against three recent state-of-the-art monocular models: DPT-Hybrid, Marigold, and GeoWizard.

Depth Anything V2 achieves the lowest AbsRel on KITTI and highly competitive performance on NYUv2, demonstrating strong generalization in indoor and outdoor domains. Although Marigold and GeoWizard show strong accuracy in indoor benchmarks, Depth Anything’s balance between inference quality and deployment practicality makes it a good candidate for real-time use.

Its open-source availability, support for various ViT backbone sizes, and robust performance under varying surface textures and lighting conditions make it especially suitable for this application. These characteristics are critical in food volume estimation tasks, where repeatability, affordability, and integration simplicity are key to success.

### 2.3. Application of the Depth Map

The depth maps generated by Depth Anything V2 are a critical component of the volume estimation pipeline. These maps provide relative depth information for every pixel within the tray region, enabling geometric reasoning over the food’s three-dimensional structure.

Unlike typical 2D-based image analysis, this approach leverages the estimated depth data to approximate the elevation of each segmented food item above the plate surface. This process involves the following:

- Isolating the relevant region of interest (ROI) based on the detected tray.
- Aligning the segmentation masks with the corresponding pixels in the depth map.
- Applying geometric operations to correct perspective-induced distortion, ensuring that all measurements are made relative to a consistent reference plane.

As the depth output is normalized and uncalibrated in metric units, additional corrections and assumptions are necessary before numerical integration. The following sections describe the core steps applied to extract meaningful volume estimations from the relative depth maps, beginning with a planar correction technique that mitigates global tilt artifacts across the tray.

### 2.4. Food Detection and Cropping

Food detection in this study relies on a YOLOv8 model [10], previously trained and validated on a custom dataset of cafeteria trays, as detailed in our earlier work [9]. The model is capable of identifying trays, plates, and a variety of food items commonly found in institutional food service environments.

In this pipeline, the primary role of the detection module is to locate the tray within each RGB frame. Once detected, the corresponding bounding box is extracted and used to crop the region of interest from the original image. This operation serves three key purposes:

1. Background removal: eliminates irrelevant visual elements that could negatively affect the performance of the monocular depth estimation model.
2. Context standardization: spatially centers the food region, improving consistency in the subsequent depth maps.
3. Segmentation guidance: provides bounding box prompts for later instance segmentation of individual food components.

**Figure 3:**
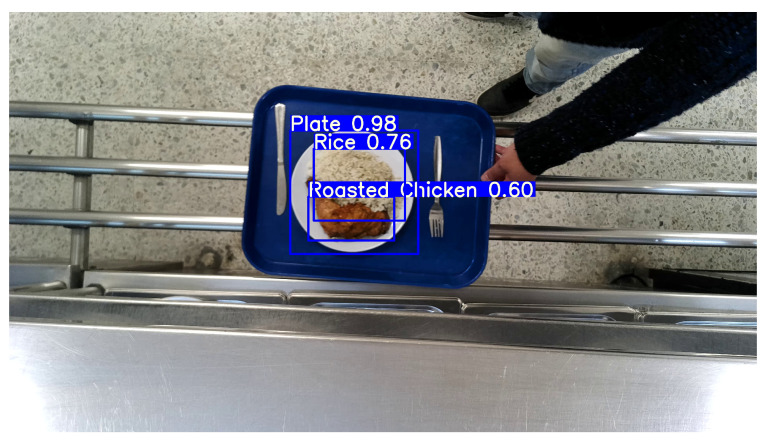
Example of food detection using YOLOv8. The system identifies the tray and labels its contents with bounding boxes.

An example of a detection output is shown in Figure 3. For visualization clarity, the figure highlights the plate and food items, although the model is also trained to detect the tray itself. In practice, the cropped region used as input for the subsequent monocular depth estimation and segmentation steps corresponds to the bounding box of the detected tray.

### 2.5. Segmentation Process

Recent advances in image segmentation have highlighted the versatility and effectiveness of the Segment Anything Model (SAM) [11]. SAM has demonstrated robust performance in various domains, including natural scenes, medical imaging, and food segmentation. In particular, studies such as FoodSAM [7] and FoodInsSeg [8] have showcased the ability of SAM to accurately segment complex food items without the need for task-specific training.

**Figure 4:**
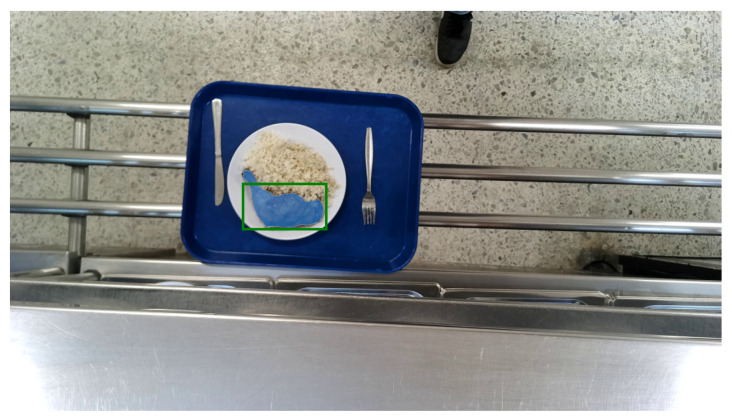
Example of food segmentation using SAM. The model accurately delineates individual food items, enabling precise volume estimation. The green bounding box indicates where the item of food is.

In our pipeline, SAM was employed to generate precise pixel-level masks for each food component, as shown in Figure 4. Utilizing bounding box prompts derived from the detection stage, SAM produced binary masks that were directly applied to the corresponding depth maps, ensuring spatial alignment. This approach offers several advantages.

1. Enhanced Boundary Precision: The ability of SAM to capture fine-grained details allows for accurate segmentation of curved or irregular food borders, which is crucial for precise volume estimation.
2. Improved Generalization: The zero-shot capabilities of SAM enable it to perform well across various food types and presentations without the need for task-specific training.
3. Streamlined Workflow: Replacing the YOLO-based segmentation model used in our previous work [9] with SAM simplifies the segmentation process, reducing the need for extensive labeled datasets.

Each segmentation mask was stored individually to facilitate subsequent analyzes, such as calculating the area, centroid positions, and assessing shape consistency across frames.

### 2.6. Base Plane Correction

One of the primary challenges observed with monocular depth estimation was the presence of an artificial slope across the tray, likely caused by the model’s interpretation of perspective cues rather than an actual geometric inclination. This tilt introduces bias into volume calculations, particularly when food items are positioned away from the center of the plate.

To counter this, a base plane correction step was introduced. The segmented plate was assumed to be flat, and the outer edge of the plate—extracted from the SAM-generated mask—was sampled to fit a regression plane using least squares. This geometric approximation assumes that the peripheral area of the plate reflects the base level.

The fitted plane is defined as

(1) z=ax+by+c

It is then subtracted from the original depth map to yield a corrected map:

(2) $$z_{\mathrm{corrected}}\left(x,y\right)=z_{\mathrm{depth}}\left(x,y\right)-\left(ax+by+c\right)$$

This correction effectively removes tilt bias and re-centers the scene to reflect the relative depth above the plate surface. Only positive values of $z_{\mathrm{corrected}}$ are retained for volume integration, corresponding to pixels lying above the estimated base plane.

**Figure 5:**
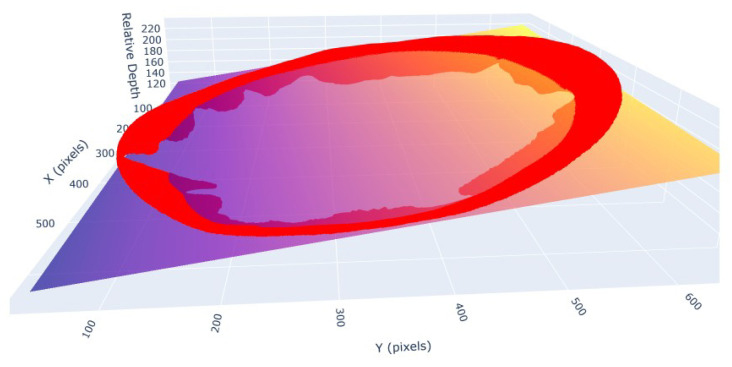
Example of corrected depth map after base plane subtraction. The artificial slope has been removed, aligning the food components to a consistent base level. The purple/yellow plane shows the inclination. The other colors just correspond to the objects on the plane.

Figure 5 illustrates the estimation of the base plane. The red points represent the sampled outer boundary of the plate, used as input for least-squares surface fitting. The resulting plane serves as a geometric reference for all subsequent depth corrections.

### 2.7. Volume Estimation

The relative volume *V* of each segmented food item was computed by summing the corrected depth values across the corresponding binary mask:

(3) $$V=\sum_{(x,y)\in \mathrm{mask}}z_{\mathrm{corrected}}\left(x,y\right)\cdot s^{2}$$

where $s^{2}$ denotes the area per pixel, and $z_{\mathrm{corrected}}\left(x,y\right)$ is the depth value at pixel (x,y) after base plane subtraction. This formulation treats each pixel as a small vertical prism, with a base area $s^{2}$ and height given by its corrected depth, and integrates these elements to approximate the total volume above the plate surface.

At this stage of development, volumes are expressed in arbitrary units due to the lack of metric calibration. However, this representation remains valid for relative comparisons, such as determining which ingredient occupies more space or for future mapping to physical units via calibration factors.

This method is highly sensitive to the accuracy of both segmentation and depth correction. Small errors in boundary delineation or residual slope in the estimated plane can accumulate over hundreds or thousands of pixels, potentially distorting the total volume. As such, validating the system with a known object and repeated measures becomes a necessary next step in assessing the reliability and precision of the estimation procedure.

The following section describes a set of controlled experiments using a calibration object and repeated food samples to evaluate the consistency of the proposed method under spatial displacement and object rotation.

### 2.8. Runtime Performance and Computational Cost

The runtime performance of the proposed pipeline was analyzed to assess its computational feasibility for practical deployment in institutional dining environments. The analysis was conducted on a representative RGB frame processed through the main stages of the pipeline, including food segmentation, monocular depth estimation, and volume computation. All experiments were executed using a Python-based implementation with PyTorch version 2.5.1 on a workstation equipped with an NVIDIA GPU [17].

Table 3 summarizes the execution time of the main processing stages and their relative contribution to the reported runtime. The computational cost is dominated by segmentation stages based on the Segment Anything Model (SAM) [11], which together account for more than 91% of the reported execution time. Food segmentation represents the largest contributor (50.7%), followed by plate border segmentation (40.6%). These operations involve high-resolution mask generation and therefore incur significant computational overhead.

Monocular depth estimation using Depth Anything V2 [6] requires approximately 1.15 s per frame, corresponding to 8.4% of the reported runtime. Despite employing a large Vision Transformer backbone (ViT-L), depth inference is not the primary bottleneck of the system. Other stages, including image loading, preprocessing, detection, and volume computation, contribute negligibly to the total runtime (below 1%), confirming that the proposed geometric correction and volumetric integration procedures are computationally efficient.

Although the current implementation does not operate at real-time video frame rates, the system is designed for near-real-time or offline cafeteria monitoring, where each tray is analyzed independently as it passes under the camera. In such scenarios, processing a single representative frame per tray is sufficient for accurate portion estimation. Potential runtime optimizations include replacing SAM with lighter segmentation models or reducing segmentation resolution, which are left for future work as the present study prioritizes robustness and estimation accuracy over processing speed.

## 3. Results

### 3.1. Robustness of Food Volume Estimation Under Rotational Variability

To evaluate the consistency of the volume estimation system under natural variations in food orientation, we conducted a controlled experiment using real plate-served meals. Two participants were instructed to walk repeatedly under the camera while carrying the same dish: a combination of rice and a chicken leg. Across eight independent passes per participant, the plate composition remained unchanged; however, the chicken was rotated or repositioned slightly at each test.

This setup simulates realistic variability in food presentation that may occur in institutional dining settings. The goal was to assess how robust the monocular depth estimation pipeline is when such presentation-induced changes are introduced.

For each pass (rotation), the chicken was segmented in each frame in which the plate appeared fully under the camera, and its volume was estimated frame by frame. Each pass, therefore, yielded a block of estimated volumes from which the following statistics were computed:

- Mean volume: average of all volume estimations for that rotation
- Standard deviation (SD): dispersion of frame-level volumes during that pass
- Coefficient of variation (CV): relative variability defined as $\mathrm{CV}=\frac{\mathrm{SD}}{\mathrm{Mean}}$

Table 4 and Table 5 report these statistics for Person 1 and Person 2, respectively.

These results show that the proposed system maintains stable volume estimations even under moderate changes in the orientation of the food. The coefficient of variation remained below 14% in all cases, with slightly higher variability observed in **Person 1**, likely due to more irregular rotation angles or occlusions. Overall, the method demonstrates good robustness to intra-class presentation differences, which is a desirable property in practical deployment scenarios.

### 3.2. Visualization of Inter-Rotation Consistency

**Figure 6:**
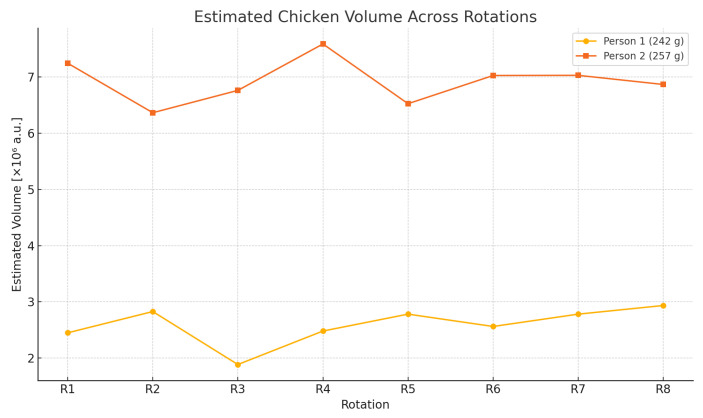
Estimated chicken volume across eight plate rotations. The participant with the heavier portion (257 g) consistently yields a higher estimated volume than the lighter one (242 g), reflecting the proportionality between mass and volume estimations.

To further analyze the effect of rotational changes on the estimated food volume, Figure 6 presents the average chicken volume estimated for each of the eight plate rotations. The values are derived from frame-level depth measurements, averaged per rotation block. The two participants used the same food composition in each trial, and their chicken portions had actual weights of 242 [g] and 257 [g], respectively.

The figure provides a visual summary of the system’s consistency and proportional response. Despite natural fluctuations in camera angle, lighting, or food orientation, the volume estimations remain stable across trials, with no erratic jumps or outlier values. Both participants exhibit minor volume deviations between passes, but the relative consistency throughout the sequence supports the reliability of the system.

Furthermore, the participant with the heavier chicken (257 [g]) consistently exhibits higher estimated volumes than the one with the lighter portion (242 [g]), in line with expectations. The nearly parallel progression of the two curves throughout all eight rotations suggests that the model preserves proportionality between mass and geometric volume, even when using relative depth values without explicit calibration.

### 3.3. Additional Generalization Test on a Different Food Item

To further evaluate the generalization capability of the proposed system beyond the chicken-based trials, we processed both the RGB frame and the corresponding monocular depth map of an additional food item not included in the rotation experiment. This complementary test served as an out-of-distribution validation, examining whether the segmentation and depth-based volume estimation remained stable when confronted with different food geometry and texture.

**Figure 7:**
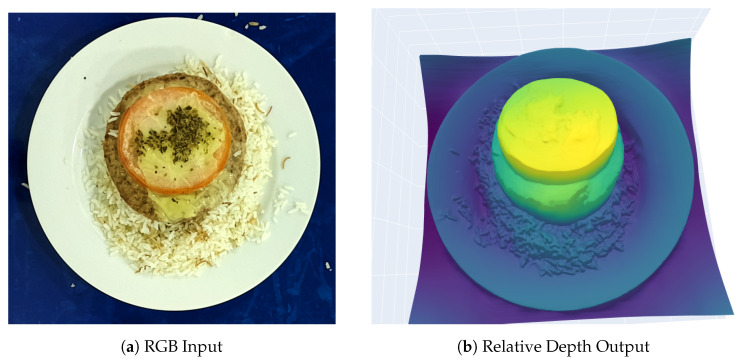
RGB image and corresponding relative depth map computed for the additional food item used in the out-of-distribution generalization test.

The resulting RGB–depth pair is shown in Figure 7. The system successfully preserved clear object boundaries and generated coherent depth gradients, enabling a consistent volume estimation for this new food item. The estimated volume was 8,166,206 arbitrary units, which aligns with the expected proportional increase relative to the chicken samples previously analyzed. These findings further support the robustness and transferability of the depth-estimation pipeline across a wider variety of served meals in institutional dining contexts.

### 3.4. Frame-Level Variability Analysis

To quantify the intra-pass consistency of the volume estimation system, we computed the coefficient of variation (CV) across *all individual frames* collected during the experiment for each participant. Unlike the inter-rotation CV reported previously, this metric captures the fluctuation of volume estimations on a frame-by-frame basis, reflecting the stability of the method during each camera traversal.

Table 6 summarizes the results. Person 2 exhibits significantly lower frame-wise variability (15.2% CV) compared to Person 1 (24.9% CV), suggesting more stable estimations under their capture conditions.

This difference may stem from several factors: smoother tray movement, more stable lighting reflections, or differences in segmentation accuracy between trials. Although the sample size of this experiment does not allow for a formal test of statistical significance, the variability observed across participants was consistent with the expected behavior of repeated vision-based measurements. These results, therefore, support the robustness of the proposed depth estimation pipeline under practical conditions.

## 4. Conclusions and Future Works

This work laid the foundation for a food distribution monitoring system within institutional or corporate cafeteria settings. Through real-world experiments, procedures for food ingredient identification and portion size estimation were developed and validated. Traditional RGB cameras, integrated with YOLO neural networks, proved effective for food identification, provided that high-quality training images were used. Portion weight estimation was achieved by measuring food volume on plates using a specialized procedure that integrates RGB and depth cameras. This procedure involves training an estimation model tailored to specific food types, such as rice and chicken, with estimation errors of 5.07% and 3.75%, respectively. Stereo cameras were also validated for their precision in distance measurement, particularly when equipped with complementary lighting patterns. The reported error is based on an analysis of volume variation, which remained consistent across repeated trials, such as rotating the plate under similar conditions. While confidence intervals or error bars were not included in this version, the consistency of measurements suggests low variability. We acknowledge that a more statistically rigorous evaluation, including multiple runs and confidence intervals, would strengthen the results. Similarly, the Coefficient of Variation (CV) analysis was intended to assess robustness under variations such as plate rotations. A comprehensive statistical validation, including *p*-values or ANOVA, would indeed be desirable but requires additional time and resources. Limitations: Despite the promising results, this study has several limitations that warrant consideration. First, the Depth Anything model relies heavily on surface texture to generate accurate depth maps, which can lead to reduced precision when food items have smooth or uniform textures. Second, estimating the correct volume for ingredients with irregular shapes—such as proteins or overlapping side dishes—remains challenging and may introduce errors in weight estimation. Third, dataset imbalance is an inherent issue, as certain ingredients are consumed more frequently than others, resulting in skewed distributions that can affect the performance of object recognition algorithms during training or fine-tuning. Addressing these limitations will be critical for improving system robustness and generalization in future iterations, and they will be explicitly considered in the next phase of development. Future work will focus on consolidating the current prototype, which was developed through a combination of MATLAB (for rapid experimentation and visualization) and Python (for deep learning and image processing with libraries such as PyTorch and OpenCV). The goal is to migrate toward a unified and production-ready implementation. In this context, Python offers rapid deployment and integration with existing AI toolchains, while C++ is being considered for scenarios that demand higher efficiency and tighter hardware integration. In parallel, we plan to evaluate cloud versus local server deployment and to develop a user-friendly reporting system to facilitate practical management. Stereo cameras were used to measure food depth, relying on its surface texture. If the texture is low, laser or IR can be an alternative due to affordability. This directly addresses the first limitation related to texture dependency. LiDAR technology, for example, enhances depth measurement accuracy, especially on smooth surfaces where other cameras face challenges. Using structured light projection can improve depth measurement precision for irregular food shapes, offering a better solution than current methods. These improvements will help mitigate the second limitation concerning irregular ingredient shapes. Data imbalance, however, can lead to potential overfitting issues. To address this, augmentation techniques are recommended for balancing datasets and overcoming these challenges. Thorough cross-validation with diverse datasets is also crucial to assess the model’s generalization to new data. These steps will directly tackle the third limitation regarding unbalanced databases. Additionally, these algorithms could support scenarios involving multiple trays by extracting data from visible trays, discarding incomplete ones, and enabling real-time tracking. Future research could explore the integration of RGB imaging with thermal and LiDAR data to enhance detection accuracy under challenging conditions, such as low-light conditions or occlusion. This multimodal approach has the potential to enhance robustness and reliability in real-world applications. However, implementing these modalities introduces practical challenges, including higher costs and the need for sensors with sufficient resolution to detect small food portions accurately. Addressing these limitations will be critical for developing scalable and efficient solutions. Additionally, future work could involve introducing random induced position errors, modeled as white noise with varying variances, to evaluate their impact on segmentation accuracy, volumetric measurements, and ultimately, weight estimation. Finally, future work could investigate alternative configurations of the three complementary subsystems: YOLO for tray, dish, and ingredient identification; SAM for pixel-level segmentation within bounding boxes; and Depth Anything for depth estimation to compute volume. Since these components are interdependent, a traditional ablation study is not feasible, as removing any module would render the system inoperable. However, a modified approach could involve systematically replacing individual components with alternative models and assessing overall performance under different combinations.

## Figures and Tables

**Table 1:** Technical specifications of the Hikvision DS-2CD2786G2-IZS RGB camera.

Parameter	Value	Description
Sensor type	1/1.8” Progressive Scan CMOS	High-quality imaging sensor
Max. resolution	3840 × 2160 pixels	8 MP resolution for detailed capture
Main stream resolutions	2688 × 1520, 1920 × 1080, etc.	Frame rate: 30 FPS
Video compression	H.265/H.265+/H.264	High-efficiency formats supported
Lens type	2.8–12 mm motorized varifocal	Adjustable FOV from 108° to 46° (H)
Aperture	F1.4	Low-light sensitivity enhancement
WDR	120 dB	Wide dynamic range for backlight scenes
IR illumination	Up to 40 m	Night vision via IR LEDs
Protection	IP66/IK10	Water, dust, and vandal resistance

**Table 2:** Quantitative comparison of monocular depth estimation models on NYU Depth v2 and KITTI datasets using Absolute Relative Error (AbsRel). Lower is better.

Model	NYU AbsRel ↓	KITTI AbsRel ↓
Depth Anything V2 (ViT-L) [6]	0.056	0.045
DPT-Hybrid [12]	0.110	0.062
Marigold [15]	0.055	0.099
GeoWizard [16]	0.052	0.097

**Table 3:** Runtime breakdown of the proposed pipeline for a single RGB frame.

Stage	Time (s)	Percentage (%)
Food segmentation (SAM)	6.97	50.7
Plate border segmentation (SAM)	5.57	40.6
Monocular depth estimation (Depth Anything V2)	1.15	8.4
Other operations	∼0.05	0.3
Total (reported)	13.74	100

**Table 4:** Estimated chicken volume and variability across eight plate rotations for Person 1. Each value is derived from frame-wise estimations during a single pass under the camera.

Rotation	Mean Volume [a.u.]	SD (%)
Rotation 1	2,448,803	12.5
Rotation 2	2,827,374	10.6
Rotation 3	1,884,739	13.7
Rotation 4	2,480,449	9.5
Rotation 5	2,781,133	10.5
Rotation 6	2,561,689	9.3
Rotation 7	2,780,916	7.6
Rotation 8	2,934,666	7.2

**Table 5:** Estimated chicken volume and variability across eight plate rotations for Person 2. Each value is derived from frame-wise estimations during a single pass under the camera.

Rotation	Mean Volume [a.u.]	CV (%)
Rotation 1	7,244,201	11.5
Rotation 2	6,364,047	10.7
Rotation 3	6,761,096	6.4
Rotation 4	7,586,902	8.3
Rotation 5	6,524,396	8.3
Rotation 6	7,026,012	8.9
Rotation 7	7,029,018	9.2
Rotation 8	6,866,476	8.2

**Table 6:** Coefficient of variation (CV) across all frames for each participant. This measures the frame-wise consistency of the estimated volume.

Participant	CV Across All Frames (%)
Person 1 (242 g)	24.9
Person 2 (257 g)	15.2

## Data Availability

Dataset available on request from the authors.
